# Home dampness and molds and occurrence of respiratory tract infections in the first 27 years of life: the Espoo Cohort Study

**DOI:** 10.1093/aje/kwaf200

**Published:** 2025-09-09

**Authors:** Joona Maaranen, Timo T Hugg, Inês Paciência, Maritta S Jaakkola, Jouni J K Jaakkola, Aino K Rantala

**Affiliations:** Center for Environmental and Respiratory Health Research (CERH), Research Unit of Population Health, University of Oulu, Oulu, Finland; Center for Environmental and Respiratory Health Research (CERH), Research Unit of Population Health, University of Oulu, Oulu, Finland; Center for Environmental and Respiratory Health Research (CERH), Research Unit of Population Health, University of Oulu, Oulu, Finland; Center for Environmental and Respiratory Health Research (CERH), Research Unit of Population Health, University of Oulu, Oulu, Finland; Center for Environmental and Respiratory Health Research (CERH), Research Unit of Population Health, University of Oulu, Oulu, Finland; Finnish Meteorological Institute, Helsinki, Finland; Center for Environmental and Respiratory Health Research (CERH), Research Unit of Population Health, University of Oulu, Oulu, Finland

**Keywords:** dampness, mold, respiratory tract infection, cohort

## Abstract

The role of residential dampness and molds in the occurrence of respiratory tract infections is not well understood. We assessed the relations between cumulative lifetime and time-specific dampness and mold exposures and the occurrence of upper and lower respiratory tract infections (URTI and LRTI) from pregnancy to 27 years of age in the prospective population-based Espoo Cohort Study (*n* = 2568). We assessed 3 questionnaire-based reports of residential exposure to water damage, moisture on the surfaces, visible mold and mold odor, and incidence rates of URTI and LRTI when children were 1-6, 7-13, and 21-27 years. We estimated adjusted incidence rate differences (aIRD) and ratios (aIRR) with their 95% CI. According to all the data combined from 3 follow-ups, home exposure to dampness and mold increased the risk of URTIs (aIRR 1.15 [95% CI, 1.10-1.21]) and LRTIs (aIRR 1.47 [95% CI, 1.21-1.79). An exposure-response pattern was observed, with each additional exposure time point particularly associated with an increased risk of LRTIs due to water damage (aIRR 2.13; 1.32-3.44) and mold odor (aIRR 2.04; 1.22-3.43). The occurrence of respiratory tract infections was associated with both presence and duration of residential dampness and mold exposure.

## Introduction

Respiratory tract infections are one of the leading causes of health problems globally.^1^ They are especially common in children, who experience an average of 4-5 episodes of upper respiratory tract infection (URTI) annually,^2^^,^^3^ and remain frequent throughout adulthood.^4^ Severe lower respiratory tract infections (LRTIs) produce a significant burden of disease, often requiring hospitalization, with an estimated incidence of 1 episode per 100 person-years in industrialized countries.^1^ Given their high occurrence, respiratory tract infections have a considerable impact on public health worldwide.

Indoor environmental factors, such as dampness and molds, have a significant impact on people’s health and may increase susceptibility to respiratory conditions, including respiratory tract infections.^5^ Globally, it is estimated that 18%-50% of buildings are affected by dampness and molds,^6^ and in Finland, recent studies have reported moisture damage in 19% of family houses.^7^ As extreme weather conditions like heavy rain episodes, storms, and floods are predicted to increase due to climate change, moisture-related damage in homes is expected to become increasingly common.

Exposure to home dampness and molds has been consistently linked to respiratory symptoms and asthma across groups and populations.^5^^,^^6^^,^^8^^-^^12^ A meta-analysis by Fisk *et al*.^9^ found that dampness and mold exposure was associated with a 30%-50% increased risk of respiratory outcomes such as cough, wheeze, asthma, and upper respiratory symptoms. However, evidence linking dampness and molds to respiratory tract infections specifically is incomplete and limited mainly to cross-sectional studies.^13^^-^^15^

To address this gap, we assessed the association between cumulative lifetime and time-specific exposure to home dampness and molds and the occurrence of URTI and LRTI from pregnancy through young adulthood, up to 27 years of age. Our prospective population-based cohort study with a long follow-up provides new knowledge into the long-term health effects of residential dampness and mold exposures.

## Methods

### Study population

This study is based on the population-based prospective Espoo Cohort study. The source population included all the children born between January 1, 1984 and March 31, 1990 who were living in Espoo, Finland, during the baseline study. In March 1991 a parent-administered baseline questionnaire was distributed to a random sample of children from the source population. The answers were returned from 2568 children, who formed the study population. In March 1997 a 6-year follow-up study was conducted, and responses were gathered from 1984 participants with a follow-up rate of 77.3% of the baseline study population. A second follow-up study was implemented in 2010-2011, and responses were obtained from 1623 participants, representing 63.2% of the original study population. The age ranges at baseline, 6-year follow-up, and 20-year follow-up were 1-6, 7-13, and 21-27 years, respectively. Details on the study population and follow-up assessments have been published elsewhere.^10^^,^^12^^,^^16^ The study protocol was approved by the Ethics Committee of Oulu University Hospital, Oulu, Finland.

### Outcome: respiratory tract infections

The outcome of interest was the amount of respiratory tract infections during the study period. Information on respiratory tract infections was based on the questionnaires at baseline, and 6- and 20-year follow-ups.^2^^,^^16^ The occurrence of respiratory tract infections during the 12 months preceding each data collection was evaluated by asking “How often did the child/you experience the following infections during the past 12 months?”. The list of options included common cold, tonsillitis, sinusitis, and otitis media (ie, URTIs), and acute bronchitis and pneumonia (ie, LRTIs).

In addition, information on all respiratory tract infections leading to hospitalization between 1984 and 1997 was extracted for each cohort member from the Finnish National Hospital Discharge Register, and information on all respiratory tract infections leading to hospitalization or an outpatient visit between 1998 and 2010 was extracted from the Care Register for Health Care. Register-based data were linked to the cohort data using the personal identification number. We extracted register data for all (*n* = 2568) members of the Espoo Cohort Study who had received the diagnosis up to December 31, 2010. The National Hospital Discharge Register included the dates and causes of all hospital admissions requiring an overnight stay that have occurred since January 1969. The Care Register for Health Care is a continuation of the National Hospital Discharge Register that includes data on hospitalization and health care visits since 1994. The International Classification of Diseases was used to code the diagnoses.

### Exposure: home dampness and mold

The environmental determinant of interest in our study was exposure to home dampness and molds. In the baseline questionnaire, we used 4 different questions to determine water damage, moisture on the surfaces, visible mold, and mold odor^10^^,^^17^: “Have you ever had water damage in your apartment?” (No; yes, during the past 12 months; yes, only earlier); “Have you ever had wet spots on the ceilings, floors, or walls of the rooms occupied in your apartment?” (No; yes, during the past 12 months; yes, only earlier); “Have you ever had visible mold in your apartment?” (No; yes, during the past 12 months; yes, only earlier); and “Have you perceived mold odor in your apartment during the past 12 months?” (No; yes, almost daily; yes, 1-3 days a week; yes, 1-3 days a month; yes, less often).

In addition to similar questions about the presence of the 4 exposure indicators, the 6-year questionnaire also included a more detailed question about the timing of exposure: at pregnancy; <1; 1-3; 4-6; 7-9; 10-12 years; and during the last 12 months. In the 20-year follow-up questionnaire, the options given were the last 12 months or over 12 months ago.

### Covariates

As potential confounders in the analyses we included the following variables: sex, family socioeconomic status at baseline, duration of breastfeeding, maternal smoking during pregnancy, and environmental tobacco smoke exposure during pregnancy and from 0 to 3 years of age. Information on the covariates was obtained from the baseline and 6-year follow-up questionnaires. Family socioeconomic status was determined by combining the highest level of parental education with the highest parental occupational level at baseline.

### Statistical analysis

We applied negative binomial regression analysis to estimate incidence rate ratio (IRR) and difference (IRD) as measures of the effect of exposure to dampness and molds on the risk of respiratory tract infection, adjusting for potential confounders in multivariable models. Incidence rates (IR) of URTIs and LRTIs were compared between exposed and unexposed individuals. For the computation of IRs based on questionnaires, each participant contributed 1 person-year to the denominator, and the number of respiratory infections reported during the last 12 months contributed to the numerator. For the computation of IRs based on hospitalization, each participant contributed to 6 person-years for the follow-up period from age 0-6; 7 person-years for the follow-up period from age 7-13; 14 person-years for the follow-up period from age 14-27 years; and 27 person-years for the follow-up period from age 0-27. Separate analyses were conducted for questionnaire-based (ie, parent or self-reported) and register-based respiratory tract infections.

Associations were assessed cross-sectionally at each follow-up and follow-up-specific estimates were pooled using meta-analysis applying the restricted maximum likelihood (REML) estimator (STATA software). In addition, we prospectively evaluated associations between exposures and outcomes by ensuring exposure assessment preceded infection onset, and by examining the effects of persistent exposure (ie, exposure at 2 or more time points) and an exposure-response relationship (ie, number of exposure points). In a more detailed 6-year follow-up analysis, exposures were inquired and categorized across 6 time periods: during pregnancy, <1, 1-3, 4-6, 7-9, and 10-12 years, and an exposure-response relationship was estimated between this cumulative exposure measure and the IR of URTIs and LRTIs.

As a sensitivity analysis, we assessed whether the association between home dampness and mold exposure and childhood respiratory infections differed by the child’s asthma status (defined at each follow-up). We evaluated multiplicative interaction by including an interaction term between dampness and mold exposure and asthma in the regression model.

Analyses were conducted using SAS, version 9.4 (SAS Institute, Inc., Cary, North Carolina).

## Results

### Characteristics of the study population

Characteristics of the study population at baseline and in 6- and 20-year follow-ups are presented in Table 1. The IR of respiratory tract infections was highest in children aged 1-6 years (baseline), compared to those aged 7-13 (6-year follow-up) and 20-27 years (20-year follow-up) (Table S1). Before age 6, children experienced an average of 380 URTIs and 28 LRTIs per 100 person-years. Children aged 7-13 years had 252 URTIs and 14 LRTIs per 100 person-years at 7-13 years, and those aged 20-27 years had 265 URTIs and 13 LRTIs per 100 person-years (Tables S1-S7 and Belachew et al.^2^).

**Table 1:** Characteristics of the study population at baseline and in 6- and 20-year follow-ups, The Espoo Cohort Study, 1991-2011.

	**Follow-up assessment**					
	**Baseline**		**6-year**		**20-year**	
**Variables**	**No.**	**%**	**No.**	**%**	**No.**	**%**
No. of subjects	2568	100	1984	77.3	1623	63.2
Age, years^a^	3.6 (1.8)		10.1 (1.8)		23.0 (1.8)	
Gender						
Female	1257	48.9	982	49.5	869	53.5
Male	1311	51.1	1002	50.5	754	46.5
Family socioeconomic status						
Low	667	26.1	498	25.2	371	22.9
Medium/High	1889	73.9	1478	74.8	1246	77.1
Duration of breastfeeding, months						
<4	481	19.3	346	18.0	289	18.3
4-7	670	26.9	511	26.5	420	26.6
≥8	1337	53.7	1071	55.5	869	55.1
Maternal smoking during pregnancy						
No	2199	85.8	1722	86.9	1415	87.4
Yes	364	14.2	260	13.1	204	12.6
Environmental smoke exposure during pregnancy						
No	2467	96.07	1883	94.91	1564	96.36
Yes	101	3.93	101	5.09	59	3.64
Environmental tobacco smoking from 0 to 3 years						
No	2269	88.36	1751	88.26	1429	88.05
Yes	299	11.64	233	11.74	194	11.95

There were missing values for family socioeconomic status (*n* = 12), duration of breastfeeding (*n* = 79), and maternal smoking (*n* = 5); percentages were calculated using the respective number of subjects at each follow-up point as the denominator.

a Values are expressed as mean (SD).

### Exposure to home dampness and molds and occurrence of respiratory tract infections

**Figure 1:**
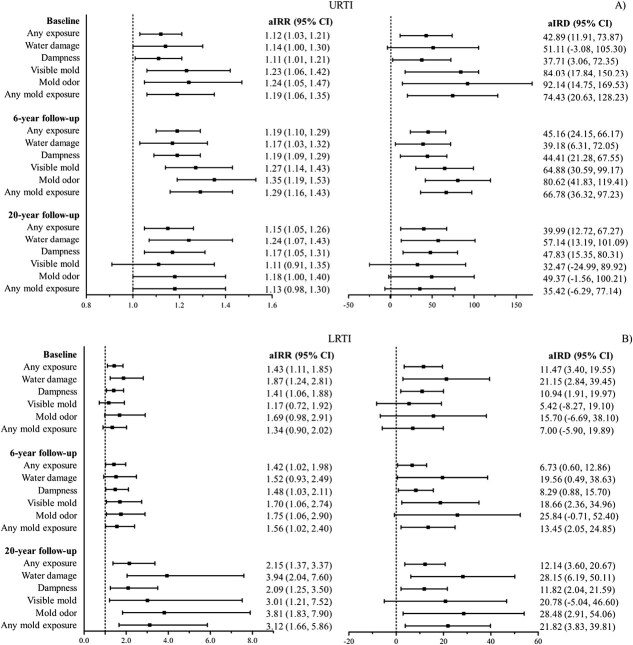
Adjusted incidence rate ratios (IRR) and incidence rate differences (IRD) with 95% CI for the association between any home dampness and mold exposure and incidence of URTI (A) and LRTI (B) from childhood to young adulthood, The Espoo Cohort Study, 1991-2011. Study participants were 1-6 years old at baseline, 7-13 years old at 6-year follow-up and 21-27 years old at 20-year follow-up.

The associations between exposure to home dampness and molds and the IRs of URTIs and LRTIs withing the previous 12 months were assessed at baseline, 6-, and 20-year follow-ups. These associations with adjusted (a) IRR and IRD are shown in Figure 1 and in Tables S2-S7.

Exposure to home dampness and molds was consistently associated with increased risks of both URTIs and LRTIs from early childhood to young adulthood (Figure 1). At baseline (ages 1-6), any previous exposure was associated with increased risk of URTIs (aIRR 1.12 [95%, CI 1.03-1.21]) and LRTIs (aIRR 1.43 [95%, CI 1.11-1.85]), after adjusting for relevant covariates. These associations corresponded to approximately 43 additional URTI episodes (aIRD 42.89 [95% CI, 11.91-73.87]) and 11 additional LRTI episodes (aIRD 11.47 [95% CI, 3.40-19.55]) per 100 person-years. Specifically, exposure to mold odor was associated with increased risk of URTIs (Figure 1), with the strongest associations observed among children exposed to mold odor monthly or more often (Tables S2 and S3).

At the 6-year follow-up (ages 7-13), any previous exposure to home dampness and molds from pregnancy to age 12 was associated with increased risks of URTIs (aIRR 1.19 [95% CI, 1.10-1.29]) and LRTIs (aIRR 1.42 [95% CI, 1.02-1.98]), which corresponded to approximately 45 additional URTI episodes (aIRD 45.16 [95% CI, 24.15-66.17]) and 7 additional LRTI episodes (aIRD 6.73 [95% CI, 0.60-12.86]) per 100 person-years (Figure 1 and Tables S4, S5). The highest increased risks of infections were observed for exposure to mold odor (Figure 1).

At the 20-year follow-up (ages 21-27), associations remained consistent. Any previous exposure to home dampness and molds was associated with a 15% increase in the risk of URTIs (aIRR 1.15 [95% CI, 1.05-1.26]) and a 115% increase in LRTIs (aIRR 2.15 [95% CI, 1.37-3.37]), corresponding to approximately 40 additional URTI episodes (aIRD 39.99 [95% CI, 12.72-67.27]) and 12 additional LRTI episodes (aIRD 12.14 [95% CI, 3.60-20.67]) per 100 person-years (Figure 1 and Tables S6, S7). Mold odor and water damage were the most prominent risk factors (Figure 1).

Pooled data from all 3 time points confirmed the overall pattern, showing that any exposure to dampness and molds increased the risk of URTIs by 15% (aIRR 1.15 [95% CI, 1.10-1.21]) and LRTIs by 47% (aIRR 1.47 [95% CI, 1.21-1.79]) (Table 2). Water damage and mold odor were the strongest risk factors of LRTIs, with adjusted IRRs of 2.16 (1.38-3.36) and 2.43 (1.32-4.47), respectively (Table 2).

**Table 2:** Pooled adjusted IRRs (95% CI) of URTIs and LRTIs according to the reported exposure to home dampness and molds from baseline to 20-year follow-up, The Espoo Cohort Study, 1991-2011.

	**Type of infection**			
**Type of exposure**	**URTI**		**LRTI**	
	**Adjusted IRR (95% CI)** ^**a**^	***P* for heterogeneity**	**Adjusted IRR (95% CI)**	***P* for heterogeneity**
Any exposure	1.15 (1.10-1.21)	.413	1.47 (1.21-1.79)	.172
Water damage	1.14 (1.06-1.23)	.392	2.16 (1.38-3.36)	.082
Dampness	1.09 (1.03-1.15)	.476	1.42 (1.19-1.70)	.277
Visible mold	1.16 (1.06-1.27)	.616	1.34 (0.81-2.21)	.111
Mold odor	1.18 (1.08-1.30)	.764	2.43 (1.32-4.47)	.056
Any mold exposure	1.14 (1.06-1.22)	.626	1.61 (0.93-2.79)	.028

Abbreviations: IRR, incidence rate ratio, URTI, upper respiratory infection, LRTI, lower respiratory infection.

a Adjusted for sex, family socioeconomic status at baseline, duration of breastfeeding, maternal smoking during pregnancy, and environmental tobacco smoke exposure during pregnancy and from 0 to 3 years of age.

In the sensitivity analysis, we observed a statistically significant interaction between any exposure to dampness and molds and asthma status on the risk of URTIs and LRTIs at baseline and at both follow-ups (*P-*values for interaction <.05). These findings indicate that the effect of dampness and mold exposure on respiratory tract infections varies according to the child’s asthma status, suggesting that asthma may modify the strength of the association.

### Cumulative exposure to home dampness and molds and occurrence of respiratory tract infections

**Figure 2:**
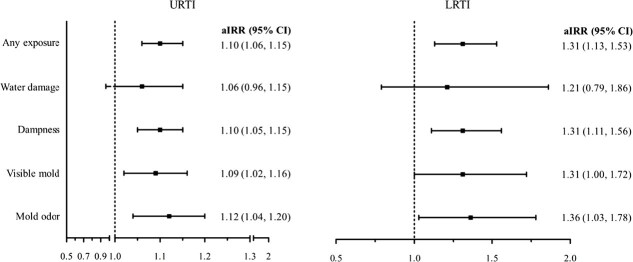
Adjusted IRR with 95% CI for the association between cumulative exposure to home dampness and molds using 6 time periods (pregnancy, <1, 1-3, 4-6, 7-9, and 10-12 years, which were specified in the 6-year follow-up survey) and incidence of URTI and LRTI, The Espoo Cohort Study, 1991-2011.

Analysis from more detailed 6-year follow-up, where exposures were categorized into 6 time periods (during pregnancy, <1, 1-3, 4-6, 7-9, and 10-12 years), showed that each additional exposure period increased the IR of URTIs by 10% (aIRR 1.10 [95% CI, 1.06-1.15]), and LRTIs by 31% (aIRR 1.31 [95% CI, 1.13-1.53]) (Figure 2). A similar exposure-response trend was observed for almost all exposure indicators.

Tables 3 and 4 present the adjusted IRRs of URTIs and LRTIs at the 6-year (age 7-13 years) and 20-year (age 21-27 years) follow-ups, respectively, according to lifetime exposure to home dampness and molds compared with the reference category (no exposure in any of the follow-ups). At the 6-year follow-up, exposures reported at that time point were more consistently associated with increased risks of URTIs and LRTIs than exposures at early life (baseline) (Table 3). Persistent exposure at both baseline and 6-year follow-up was associated with stronger associations for LRTIs, especially related to water damage (aIRR 1.89 [95% CI, 0.78-4.55]) and moisture on the surfaces (aIRR 1.93 [95% CI, 1.12-3.34]). Persistent mold exposure at both time points was not significantly associated with URTIs or LRTIs.

**Table 3:** Adjusted IRRs with 95% CI of URTIs and LRTIs at 6-year follow-up (age 7-13 years) according to lifetime exposure to home dampness and molds, The Espoo Cohort Study, 1991-2011.

		**URTIs at 6-year follow-up**	**LRTIs at 6-year follow-up**
**Exposure**	**Number**	**Adjusted IRR (95% CI)** ^**a**^	**Adjusted IRR (95% CI)** ^**a**^
No exposure (ref)	1200	1	1
Any exposure			
At baseline	492	1.03 (0.93-1.27)	1.06 (0.70-1.60)
At 6 years	579	1.18 (1.09-1.28)	1.30 (0.93-1.81)
Both	199	1.08 (0.95-1.22)	1.72 (1.07-2.77)
Water damage			
At baseline	144	1.02 (0.86-1.20)	1.08 (0.56-2.12)
At 6 years	186	1.16 (1.03-1.31)	1.38 (0.85-2.26)
Both	40	1.12 (0.87-1.44)	1.89 (0.78-4.55)
Moisture on the surfaces			
At baseline	371	1.01 (0.90-1.12)	1.08 (0.69-1.71)
At 6 years	461	1.17 (1.07-1.28)	1.35 (0.94-1.93)
Both	138	1.13 (0.97-1.30)	1.93 (1.12-3.34)
Visible mold			
At baseline	109	0.99 (0.83-1.19)	0.54 (0.20-1.45)
At 6 years	213	1.26 (1.12-1.41)	1.58 (0.98-2.54)
Both	38	0.86 (0.64-1.14)	0.52 (0.11-2.43)
Mold odor			
At baseline	82	0.97 (0.77-1.22)	0.77 (0.27-2.18)
At 6 years	174	1.33 (1.17-1.51)	1.61 (0.98-2.67)
Both	20	1.01 (0.70-1.47)	1.00 (0.19-5.25)
Any mold exposure			
At baseline	162	0.97 (0.83-1.13)	0.65 (0.30-1.40)
At 6 years	278	1.27 (1.14-1.41)	1.44 (0.94-2.22)
Both	51	0.88 (0.69-1.13)	0.56 (0.16-1.98)

Abbreviations: IRR, incidence rate ratio, URTI, upper respiratory infection, LRTI, lower respiratory infection.

a Adjusted for sex, family socioeconomic status at baseline, duration of breastfeeding, maternal smoking during pregnancy, and environmental tobacco smoke exposure during pregnancy and from 0 to 3 years of age.

**Table 4:** Adjusted IRRs with 95% CI of URTIs and LRTIs at 20-year follow-up (age 21-27 years) according to lifetime exposure to home dampness and molds, The Espoo Cohort Study, 1991-2011.

		**URTIs at 20-year follow-up**	**LRTIs at 20-year follow-up**
**Exposure**	**Number**	**Adjusted IRR (95% CI)** ^**a**^	**Adjusted IRR (95% CI)** ^**a**^
No exposure (ref)	624	1	1
Any exposure			
Baseline	492	1.14 (1.02-1.27)	1.19 (0.66-2.14)
6-y	579	1.00 (0.91-1.11)	1.18 (0.70-1.99)
20-y	328	1.23 (1.10-1.37)	2.54 (1.49-4.35)
All	29	0.99 (0.75-1.30)	0.90 (0.22-3.61)
Per 1 time point		1.05 (0.99-1.11)	1.26 (0.95-1.66)
Water damage			
Baseline	144	1.07 (0.89-1.29)	2.24 (0.97-5.14)
6-y	186	0.92 (0.79-1.07)	1.52 (0.74-3.14)
20-y	116	1.33 (1.13-1.56)	5.56 (2.61-11.82)
All	2	1.42 (0.58-3.46)	NA
Per 1 time point		1.05 (0.96-1.15)	2.13 (1.32-3.44)
Moisture on the surfaces			
Baseline	371	1.05 (0.93-1.19)	0.89 (0.45-1.78)
6-y	461	0.99 (0.89-1.11)	1.25 (0.72-2.16)
20-y	222	1.25 (1.11-1.42)	2.36 (1.30-4.28)
All	9	0.86 (0.52-1.42)	NA
Per 1 time point		1.05 (0.98-1.12)	1.19 (0.86-1.65)
Visible mold			
Baseline	109	1.21 (1.00-1.48)	1.04 (0.35-3.13)
6-y	213	1.02 (0.88-1.18)	0.79 (0.36-1.72)
20-y	59	1.21 (0.98-1.50)	4.41 (1.59-12.24)
All	1	NA	NA
Per 1 time point		1.09 (0.99-1.21)	1.40 (0.79-2.37)
Mold odor			
Baseline	82	1.13 (0.90-1.43)	0.78 (0.20-3.02)
6-y	174	1.06 (0.91-1.23)	1.99 (0.98-4.04)
20-y	82	1.31 (1.09-1.57)	4.68 (2.03-10.79)
All	2	0.64 (0.21-1.97)	1.21 (0.03-44.79)
Per 1 time point		1.10 (1.00-1.22)	2.04 (1.22-3.43)
Any mold exposure			
Baseline	162	1.21 (1.02-1.42)	0.90 (0.35-2.29)
6-y	278	1.04 (0.92-1.18)	1.42 (0.75-2.66)
20-y	119	1.22 (1.04-1.43)	3.60 (1.76-7.38)
All	4	0.65 (0.26-1.63)	0.84 (0.03-20.22)
Per 1 time point		1.08 (1.00-1.18)	1.52 (1.00-2.31)

Abbreviations: IRR, incidence rate ratio; NA, not applicable; URTI, upper respiratory infection; LRTI, lower respiratory infection.

a Adjusted for sex, family socioeconomic status at baseline, duration of breastfeeding, maternal smoking during pregnancy, and environmental tobacco smoke exposure during pregnancy and from 0 to 3 years of age.

At the 20-year follow-up, exposures reported at that point showed the strongest associations with URTIs and LRTIs for all 4 exposure indicators (Table 4). The risk of LRTIs was especially increased with exposure to water damage (aIRR 5.56 [2.61-11.82]) and mold odor (aIRR 4.68 [2.03-10.79]). Past exposures at earlier follow-ups showed weaker or nonsignificant associations with infections in young adulthood. Persistent exposure across all time points was rare and does not show consistent associations possibly due to low power (Table 4). When exposures were analyzed per 1 time point (indicating an exposure-response pattern), there was a modestly increased risk of URTIs for mold odor (IRR 1.10 [95% CI, 1.00-1.22]). For LRTIs, the associations were stronger, particularly for water damage (IRR 2.13 [95% CI, 1.32-3.44]) and mold odor (IRR 2.04 [95% 1.22-3.42]) (Table 4).

### Exposure to home dampness and molds and occurrence of URTIs and LRTIs based on the hospitalization register

We also investigated the associations of home exposure with the incidence of URTI and LRTIs requiring hospitalization or health care visit (Tables S8 and S9). Although the number of cases in each group was small, we found that those participants who were exposed to dampness and molds at home during childhood experienced 30 URTIs per 100 person-year by age 27 years compared with 20 URTIs in those who were not exposed (aIRD 13.08 [95% CI, 2.90-23.25] and aIRR 1.71 [95% CI, 1.22-2.41]).

## Discussion

### Main findings

Based on our 20-year prospective cohort study, both the occurrences of URTIs and LRTIs from childhood to young adulthood were consistently associated with exposure to home dampness and molds. Pooled analysis from the baseline, 6-, and 20-year follow-ups indicated that any exposure to dampness and molds increased the risk of URTIs by 15% and LRTIs by 47%. Among the exposure indicators, reported mold odor at home appeared to be the most harmful factor for respiratory tract infections. Previous exposure to mold odor was associated with a 24%-35% increased risk of URTIs and a 69%-75% increased risk of LRTIs in preschool-aged (baseline) and school-aged (at 6-year follow-up) children, and up to an 18% increase in URTIs and a 281% increase in LRTIs in young adulthood (20-year follow-up).

Our findings also suggest that the occurrence of respiratory tract infections is most strongly associated with current exposure to dampness and mold than past exposure, emphasizing the strong impact of ongoing exposure. However, persistent exposure (at baseline and 6-year follow-up) to water damage and moisture on surfaces was associated with more pronounced effects for LRTIs. A clear exposure-response relationship related to water damage and mold odor was observed, suggesting the cumulative risk of repeated exposure.

Finally, our registry-based data confirmed that childhood exposure to any form of dampness and mold at baseline was associated with a 70% higher risk of URTIs requiring a medical visit by age 27, whereas the register-based analysis did not show any significant association for LRTI.

### Synthesis with previous knowledge

According to a recent systematic review and meta-analyses, there is evidence on associations between residential dampness and mold exposure and respiratory tract infections and related symptoms in children in high-income countries.^15^ However, the review also highlighted a lack of prospective cohort studies examining the long-term impact of dampness and molds on the burden of respiratory tract infections.^15^ Based on their meta-analyses, which primarily included cross-sectional studies, exposure to dampness and mold increased the risk of any respiratory tract infections (OR 1.28 [95% CI, 1.08-1.53] and OR 1.59 [95% 1.12-2.25], respectively). Consistently, our results demonstrate that exposure to dampness significantly increased the risk of URTIs and LRTIs with aIRRs ranging from 1.1 to 1.4, and exposure to molds with IRRs between 1.1 and 1.6 at any time during the first 27 years of life. Additionally, a recent large cross-sectional study of over 42 000 children from the 11-year follow-up of the Danish National Birth Cohort, reported similar associations: residential dampness and mold exposure were linked to increased rates of common colds, influenza, and tonsillitis in the past year.^18^

While there have been some studies on dampness and mold exposures and respiratory tract infections in children^13^^,^^18^^,^^19^ there have been fewer studies on the associations with adults, except for one among Finnish university students.^14^ That study, involving 10 000 Finnish university students between the ages of 18 and 25, reported an association between home dampness and the occurrence of frequent common colds.^14^ Based on their findings, visible mold, damp stains, or water damage were associated with frequent common colds and other respiratory tract infections with adjusted OR of 1.28 (95% CI, 1.09-1.47). To address the gap of knowledge on adults, our prospective study followed the study population until the age of 20-27 and found similar results, showing that exposure to dampness and molds increased the incidence of URTI and LRTI.

A case-control study among children under the age of 2 in New Zealand, comprising 188 cases and 454 controls, provided evidence of a dose-response relationship between housing quality measures, such as dampness and molds, and acute respiratory illness hospitalization rates in young children^13^. Our study also confirms the dose-dependent increase in infection risk by showing that each additional exposure time period increases the risk of infections.

Recent research on this topic has focused on the role of different mold species in causing respiratory symptoms.^8^^,^^20^ A study conducted in Finnish and Dutch schools found that bacteria growing in damp environments may play a bigger role in respiratory symptoms than mold growth itself.^20^ However, mold growth still affects respiratory tract symptoms, as some mold species were detected to cause symptoms while others seemed to reduce the risk.^20^ The focus on their study was on respiratory tract symptoms, not respiratory infections, so the results do not directly correlate with the relationship between mold exposure and respiratory tract infections. Overall, based on the previous meta-analysis and our prospective study, there is increasing evidence that exposure to dampness and molds increases the risk of URTIs and LRTIs, in addition to just respiratory tract symptoms and asthma.^15^

### Validity of results

This study has strong methodological validity, as it examined the association between home dampness and mold exposure with respiratory tract infections across lifetime using a population-based cohort with prospective follow-up from early childhood into young adulthood. The relatively high response rates at baseline (80.3%), 6-year (77.3%), and 20-year (63.2%) follow-ups strengthen the study’s representativeness and validity. Moreover, the lack of substantial differences between the baseline population and those who participated in follow-ups reduces the risk of selection bias (Table 1). We were also able to control for some known risk factors for respiratory tract infections, such as exposure to environmental tobacco smoke.

Information on exposure and outcome indicators was collected through parental and self-administered questionnaires.^2^^,^^16^ Reporting in the questionnaires may have led to some misclassification, which is a limitation in our study. Parental reporting may be influenced by the health status of a child, that is, parents whose children have respiratory tract infections might be more likely to report dampness and mold exposure, potentially leading to an overestimation of associations. However, this type of reporting bias is less likely in early data collection, which occurred in 1991 before general awareness of health risks related to indoor dampness and molds became widespread. In contrast, greater awareness during follow-up assessments may have introduced differential reporting.

Importantly, the study’s findings were supported by register-based outcome data from the National Hospital Discharge Register, providing comprehensive information on respiratory tract infections requiring hospitalization from birth to age 27. Using the register data, we were able to include respiratory tract infection diagnoses from birth to age 27, whereas in the questionnaire analyses we were likely unable to capture infections that occurred outside the data collection periods. A possible misclassification would also occur if parents whose children have respiratory symptoms due to noninfectious causes (eg, allergies) misreported them as infections, but this is unlikely to be systematic with respect to home exposures.

### Biological plausibility

Damp and warm indoor conditions create an optimal environment for mold growth and chemical reactions. Under these conditions, chemical decomposition reactions in building materials can start, resulting in the release of various primary and secondary compounds (emissions) into the air.^21^^,^^22^ Repeated and prolonged exposure to chemical components and spores, particles, and metabolic products produced by molds found in damp indoor spaces can induce inflammatory reactions, suppress immune response, and therefore predisposes the body to the harmful effects of microbes.^23^ These effects may be mediated by irritation, toxicity, and allergy reactions, as well as olfactory, psychological, and neuropsychological pathways.^24^^,^^25^

The lower respiratory tract is likely more susceptible to microbes and potential other stressors than the upper respiratory tract due to factors such as critical function in gas exchange, constant immunological pressure, microbial migration, and distinct microbial diversity.^26^^,^^27^ The spores (<5 μm in aerodynamic diameter) and other submicronic fragments^28^ as well as gaseous compounds produced by molds and chemical decomposition can effectively penetrate the lower respiratory tract and potentially cause reactions. A perceived mold odor may indicate current or past mold and bacteria growth as well as the presence of chemical compounds such as microbial volatile organic compounds and mycotoxins. This odor can indicate both prolonged and intense exposure.^29^^,^^30^ This, together with the good penetration capacity of spores, submicronic fragments, and gaseous compounds, may explain our observations of a higher risk of respiratory tract infections, especially related to mold odor.

## Conclusions

To our knowledge, this is the first longitudinal study to examine the association between home dampness and mold exposure and respiratory tract infections with a follow-up extending from childhood into adulthood. Our 20-year prospective cohort study provides evidence that the occurrence of respiratory tract infection is related to both the presence and duration of exposure to residential dampness and molds. The results indicate that the adverse health effects of home dampness and molds begin in early life and persist into adulthood, including URTIs and LRTIs. The findings suggest that efforts to prevent and reduce exposure to dampness and molds in buildings would have important public health benefits. Future research should aim to objectively measure exposure and investigate the mechanisms that link dampness and molds to human immune-mediated pathways underlying respiratory problems.^23^ In addition, specific respiratory pathogens such as respiratory syncytial virus or influenza were not specifically addressed in this study, highlighting a need for further research to determine whether dampness and mold exposure differentially affects the risk of these and other distinct pathogens.

## Supplementary Material

Web_Material_kwaf200

## Data Availability

Data are available upon reasonable request from the Principal Investigator of the Espoo Cohort Study, Professor Jouni Jaakkola, E-mail: jouni.jaakkola@oulu.fi.
