# Correction to: Home dampness and molds and occurrence of respiratory tract infections in the first 27 years of life: the Espoo Cohort Study

**DOI:** 10.1093/aje/kwag148

**Published:** 2026-07-01

This is a correction to: Joona Maaranen, Timo T Hugg, Inês Paciência, Maritta S Jaakkola, Jouni J K Jaakkola, Aino K Rantala, Home dampness and molds and occurrence of respiratory tract infections in the first 27 years of life: the Espoo Cohort Study, *American Journal of Epidemiology*, Volume 194, Issue 12, December 2025, Pages 3492–3500, https://doi.org/10.1093/aje/kwaf200.

In the originally published version of this manuscript, the `**Funding**' section was incomplete.

This has been corrected to read:

``This work was funded by the Academy of Finland (grant no. 310371 and no. 310372); and the European Union (InChildHealth grant number 101056883). Views and opinions expressed are however those of the authors only and do not necessarily reflect those of the European Union or European Health and Digital Executive Agency (HaDEA). Neither the European Union nor the granting authority can be held responsible for them''.

 Instead of: 

``This work was supported by the Academy of Finland (grant no. 310371 and no. 310372); and the European Union Horizon Europe (Grant agreement no: 101056883)''. 

