# Supplementary material for: Home dampness and molds and occurrence of respiratory tract infections in the first 27 years of life: the Espoo Cohort Study
Source: Am J Epidemiol. 2025 Sep 9;194(12):3492–500. doi: 10.1093/aje/kwaf200 (PMC12671967; doi:10.1093/aje/kwaf200)
Supplement: Web_Material_kwaf200 [file web_material_kwaf200.zip › Supplementary material_Maaranen J et al._010925.docx]

**Supplementary material**

**Home dampness and molds and occurrence of respiratory tract infections in the first 27 years of life: The Espoo Cohort Study**

Joona Maaranen, Timo T. Hugg , Inês Paciência , Maritta S. Jaakkola, Jouni J.K. Jaakkola, Aino K. Rantala

Contents:

[Number of parental reported respiratory tract infections, Table S1 2](#_Toc206529994)

[Home dampness and molds and respiratory tract infections at baseline, Tables S2-S3 3](#_Toc206529995)

[Home dampness and molds and respiratory tract infections at 6-year follow-up, Tables S4-S5 5](#_Toc206529996)

[Home dampness and molds and respiratory tract infections at 20-year follow-up, Tables S6-S7 9](#_Toc206529997)

[Home dampness and molds and respiratory tract infections based on the hospitalization register, Tables S8-S9 11](#_Toc206529998)

# Number of parental reported respiratory tract infections

**Table S1. Number of upper (URTI) and lower (LRTI) respiratory tract infections in the last 12 months at baseline, 6-year follow-up, and 20-year follow-up, The Espoo Cohort Study, 1991-2011**

| **Infection** | **At baseline (1-6 years of age)** | **6-year follow-up (7-13 years of age)** | **20-year follow-up (20-27 years of age)** |
| --- | --- | --- | --- |
| **URTI** |  |  |  |
| Mean number of episodes (SD) | 3.80 (3.31) | 2.52 (3.05) | 2.65 (2.25) |
| Maximum number of episodes | 37 | 19 | 20 |
| Participants with 0 episodes | 143 | 185 | 124 |
| Participants with 1 episode | 402 | 492 | 397 |
| Participants with 2 episodes | 541 | 524 | 397 |
| Participants with ≥3 episodes | 1,482 | 773 | 635 |
|  |  |  |  |
| **LRTI** |  |  |  |
| Mean number of episodes (SD) | 0.28 (0.80) | 0.14 (0.44) | 0.13 (0.53) |
| Maximum number of episodes | 11 | 5 | 10 |
| Participants with 0 episodes | 2,111 | 1,767 | 1,420 |
| Participants with 1 episode | 322 | 146 | 91 |
| Participants with 2 episodes | 77 | 32 | 30 |
| Participants with ≥3 episodes | 58 | 8 | 12 |

SD; standard deviation

# Home dampness and molds and respiratory tract infections at baseline

**Table S2. Incidence rates (IR) per 100 person-years (PY), incidence rate differences (IRDs) and incidence rate ratios (IRRs) with 95% confidence intervals (CI) of upper respiratory tract infections in the previous 12 months according to the reported exposure to home dampness and molds at baseline (before 6 years of age), The Espoo Cohort Study, 1991-2011.**

| **Type of exposure** | **Number (%)** | **IR/100 PY** | **unadjusted IRD (95% CI)** | **adjusted IRD (95% CI)^a^** | **unadjusted IRR (95% CI)** | **adjusted IRR (95% CI)^a^** |
| --- | --- | --- | --- | --- | --- | --- |
| Total N | 2,568 | 380.18 |  |  |  |  |
| No exposure | 2,072 (80.1) | 370.99 | 1 (reference) | 1 (reference) | 1 (reference) | 1 (reference) |
| **Any exposure** | **492 (19.2)** | **416.26** | **45.27 (14.09, 76.45)** | **42.89 (11.91, 73.87)** | **1.12 (1.04, 1.21)** | **1.12 (1.03, 1.21)** |
| **Water damage** | **144 (5.6)** | **429.17** | **58.17 (3.06, 113.28)** | **51.11 (-3.08, 105.30)** | **1.16 (1.02, 1.32)** | **1.14 (1.00, 1.30)** |
| Last 12 months | 42 | 435.71 | 64.72 (-36.23, 165.67) | 69.79 (-30.43, 170.00) | 1.17 (0.93, 1.48) | 1.18 (0.94, 1.49) |
| Over 12 months ago | 102 | 426.47 | 55.48 (-9.31, 120.27) | 43.64 (-19.64, 106.92) | 1.15 (0.99, 1.34) | 1.13 (0.97, 1.31) |
| **Dampness** | **371 (14.5)** | **407.01** | **36.01 (1.47, 70.56)** | **37.71 (3.06, 72.35)** | **1.10 (1.01, 1.20)** | **1.11 (1.01, 1.21)** |
| Last 12 months | 211 | 427.88 | 56.49 (10.46, 102.53) | 60.19 (13.85, 106.53) | 1.15 (1.03, 1.29) | 1.16 (1.04, 1.30) |
| Over 12 months ago | 160 | 380.00 | 9.01 (-38.88, 56.89) | 6.97 (-40.79, 54.72) | 1.02 (0.90, 1.16) | 1.02 (0.90, 1.16) |
| **Visible mold** | **109 (4.3)** | **455.96** | **84.97 (19.14,150.80)** | **84.03 (17.84, 150.23)** | **1.23 (1.06, 1.42)** | **1.23 (1.06, 1.42)** |
| Last 12 months | 75 | 462.67 | 91.67 (11.69, 171.66) | 97.96 (17.30, 178.63) | 1.25 (1.05, 1.48) | 1.26 (1.06, 1.50) |
| Over 12 months ago | 34 | 441.18 | 70.18 (-42.96, 183.33) | 47.82 (-65.72, 161.37) | 1.19 (0.92, 1.54) | 1.15 (0.89, 1.49) |
| **Mold odor** | **82 (3.2)** | **475.61** | **104.62 (36.80, 182.43)** | **92.14 (14.75, 169.53)** | **1.28 (1.09, 1.51)** | **1.24 (1.05, 1.47)** |
| Once or more per month | 38 | 578.95 | 207.95 (74.44, 341.46) | 181.13 (50.50, 311.76) | 1.56 (1.24, 1.97) | 1.47 (1.16, 1.87) |
| Less than once a month | 44 | 386.36 | 15.37 (-74.32, 105.06) | 15.52 (-75.49, 106.53) | 1.04 (0.83, 1.31) | 1.04 (0.82, 1.31) |
| **Any mold exposure** | **162 (6.3)** | **451.23** | **80.24 (26.33, 134.15)** | **74.43 (20.63, 128.23)** | **1.22 (1.08, 1.37)** | **1.19 (1.06, 1.35)** |

Abbreviations: CI, confidence interval, IRD, incidence rate difference; IRR, incidence rate ratio, URTI, upper respiratory infection; PY, person-years

^a^Adjusted for sex, family socioeconomic status at baseline, duration of breastfeeding, maternal smoking during pregnancy, and environmental tobacco smoke exposure during pregnancy and from 0 to 3 years of age.

**Table S3. Incidence rates (IR) per 100 person-years (PY), incidence rate differences (IRDs) and incidence rate ratios (IRRs) with 95% confidence intervals of lower respiratory tract infections (LRTI) in the previous 12 months according to the reported exposure to home dampness and molds baseline (before 6 years of age), The Espoo Cohort Study, 1991-2011.**

| **Type of exposure** | **Number (%)** | **IR/100py** | **unadjusted IRD (95% CI)** | **adjusted IRD (95% CI)^a^** | **unadjusted IRR (95% CI)** | **adjusted IRR (95% CI)^a^** |
| --- | --- | --- | --- | --- | --- | --- |
| Total N | 2,568 | 28.19 |  |  |  |  |
| No exposure | 2,072 (80.1) | 26.45 | 1 (reference) | 1 (reference) | 1 (reference) | 1 (reference) |
| **Any exposure** | **492 (19.2)** | **35.57** | **9.12 (0.58, 17.67)** | **11.47 (3.40, 19.55)** | **1.34 (1.04, 1.73)** | **1.43 (1.11, 1.85)** |
| **Water damage** | **144 (5.6)** | **47.92** | **21.47 (2.39, 40.55)** | **21.15 (2.84, 39.45)** | **1.81 (1.20, 2.73)** | **1.87 (1.24, 2.81)** |
| Last 12 months | 42 | 40.47 | 14.03 (-16.043, 44.49) | NA | 1.53 (0.72, 3.27) | 1.39 (0.66, 2.92) |
| Over 12 months ago | 102 | 50.98 | 24.53 (0.66, 48.41) | 26.07 (3.73, 48.40) | 1.93 (1.19, 3.11) | 2.08 (1.29, 3.35) |
| **Dampness** | **371 (14.5)** | **34.50** | **8.05 (-1.40, 17,51)** | **10.94 (1.91, 19.97)** | **1.30 (0.98, 1.73)** | **1.41 (1.06, 1.88)** |
| Last 12 months | 211 | 37.91 | 11.47 (-1.66, 24.59) | 12.68 (0.24, 25.11) | 1.43 (1.00, 2.05) | 1.53 (1.08, 2.18) |
| Over 12 months ago | 160 | 30.00 | 3.55 (-9.17, 16.28) | 3.69 (-6.06, 13.45) | 1.13 (0.74, 1.74) | 1.26 (0.82, 1.93) |
| **Visible mold** | **109 (4.3)** | **33.03** | **6.58 (-9.61, 22.77)** | **5.42 (-8.27, 19.10)** | **1.25 (0.76, 2.05)** | **1.17 (0.72, 1.92)** |
| Last 12 months | 75 | 34.67 | 8.22 (-12.07, 28.51) | NA | 1.31 (0.73, 2.36) | 1.23 (0.68, 2.23) |
| Over 12 months ago | 34 | 29.41 | 2.96 (-23.41, 29.34) | 9.96 (-29.23, 49.16) | 1.11 (0.45, 2.73) | 1.04 (0.43, 2.52) |
| **Mold odor** | **82 (3.2)** | **45.12** | **18.67 (-5.52, 42.87)** | **15.70 (-6.69, 38.10)** | **1.71 (0.99, 2.94)** | **1.69 (0.98, 2.91)** |
| Once or more per month | 38 | 81.58 | 55.13 (-2.44, 112.70) | 50.09 (-1.93, 102.10) | 3.08 (1.51, 6.30) | 3.04 (1.50, 6.14) |
| Less than once a month | 44 | 13.64 | -12.81 (-26.58, 0.96) | -4.55 (-21.04, 11.93) | 0.52 (0.19, 1.39) | 0.55 (0.20, 1.47) |
| **Any mold exposure** | **162 (6.3)** | **36.42** | **9.97 (-4.58, 24.52)** | **7.00 (-5.90, 19.89)** | **1.39 (0.92, 2.07)** | **1.34 (0.90, 2.02)** |

Abbreviations: CI, confidence interval, IRD, incidence rate difference; IRR, incidence rate ratio, LRTI, lower respiratory infection; NA, not applicable; PY, person-years

^a^Adjusted for sex, family socioeconomic status at baseline, duration of breastfeeding, maternal smoking during pregnancy, and environmental tobacco smoke exposure during pregnancy and from 0 to 3 years of age.

# Home dampness and molds and respiratory tract infections at 6-year follow-up

**Table S4. Incidence rates (IR) per 100 person-years (PY), incidence rate differences (IRDs) and incidence rate ratios (IRRs) with 95% confidence intervals of upper respiratory tract infections in the previous 12 months according to the reported exposure to home dampness and molds at 6-year follow-up (before 13 years of age), The Espoo Cohort Study, 1991-2011.**

| **Type of exposure** | **Number (%)** | **IR/100 PY** | **unadjusted IRD (95% CI)** | **adjusted IRD (95% CI)^a^** | **unadjusted IRR (95% CI)** | **adjusted IRR (95% CI) ^a^** |
| --- | --- | --- | --- | --- | --- | --- |
| Total N | 1984 | 251.62 |  |  |  |  |
| **No exposure** | **1395** | **237.10** | 1 (reference) | 1 (reference) | 1 (reference) | 1 (reference) |
| **Any exposure** | **579 (29.3)** | **284.90** | **47.79 (26.98, 68.60)** | **45.16 (24.15, 66.17)** | **1.20 (1.11, 1.30)** | **1.19 (1.10, 1.29)** |
| During last 12 mo | 224 (11.4) | 297.76 | 60.65 (28.62, 92.69) | 60.11 (27.18, 93.04) | 1.26 (1.12, 1.40) | 1.25 (1.12, 1.40) |
| At pregnancy | 48 (2.4) | 314.48 | 77.48 (8.45, 146.51) | 68.38 (-1.15, 137.92) | 1.33 (1.06, 1.66) | 1.28 (1.03, 1.61) |
| < 1 years | 67 (3.4) | 308.96 | 71.85 (13.90, 129.80) | 68.45 (10.61, 126.28) | 1.30 (1.08, 1.58) | 1.30 (1.07, 1.57) |
| 1-3 years | 126 (6.4) | 300.00 | 62.90 (20.86, 104.93) | 63.42 (20.80, 106.03) | 1.27 (1.10, 1.46) | 1.26 (1.09, 1.45) |
| 4-6 years | 217 (11.0) | 321.20 | 84.09 (49.80, 118.39) | 82.97 (48.37, 117.57) | 1.45 (1.21, 1.51) | 1.35 (1.21, 1.51) |
| 7-9 years | 220 (11.1) | 295.41 | 58.31 (26.44, 90.18) | 53.57 (21.63, 85.51) | 1.25 (1.12, 1.39) | 1.23 (1.10, 1.37) |
| 10-12 years | 95 (4.8) | 285.11 | 48.00 (1.87, 94.14) | 45.57 (-0.78 91.92) | 1.20 (1.02, 1.42) | 1.20 (1.02, 1.42) |
| cumulative exposure**^b^** |  |  | 30.49 (17.73, 43.25) | 28.59 (15.77, 41.40) | 1.11 (1.07, 1.15) | 1.10 (1.06, 1.15) |
| **Water damage, all** | **186 (9.5)** | **282.16** | **45.06 (11.75, 78.36)** | **39.18 (6.31, 72.05)** | **1.19 (1.06, 1.34)** | **1.17 (1.03, 1.32)** |
| During last 12 mo | 52 (2.7) | 301.92 | 64.82 (1.04, 128.60) | 59.98 (-3.24, 123.20) | 1.27 (1.03, 1.58) | 1.24 (1.01, 1.54 |
| At pregnancy | 8 (0.4) | 250.00 | 12.90 (-128.48, 154.27) | 14.32 (-127.16, 155.80) | 1.05 (0.60, 1.86) | 1.04 (0.59, 1.82) |
| < 1 years | 13 (0.7) | 184.62 | -52.49 (-143.05, 38.07) | -32.14 (-120.38, 56.09) | 0.78 (0.48, 1.27) | 0.82 (0.50, 1.34) |
| 1-3 years | 32 (1.6) | 234.38 | 2.73 (-70.76, 65.30) | 3.71 (-63.98, 71.39) | 0.99 (0.74, 1.31) | 0.99 (0.74, 1.33) |
| 4-6 years | 65 (3.3) | 304.62 | 67.51 (9.75, 125.27) | 58.55 (1.38, 115.72) | 1.28 (1.06, 1.56) | 1.26 (1.04, 1.52) |
| 7-9 years | 51 (2.6) | 290.20 | 53.09 (-9.67, 115.85) | 52.19 (-10.33, 114.72) | 1.22 (0.98, 1.52) | 1.22 (0.98, 1.52) |
| 10-12 years | 23 (1.2) | 282.61 | 45.50 (-45.33, 136.34) | 31.36 (-56.97, 119.70) | 1.19 (0.86, 1.65) | 1.14 (0.82, 1.59) |
| cumulative exposure**^b^** |  |  | 16.80 (-9.58, 43.19) | 14.47 (-11.02, 39.96) | 1.06 (0.97, 1.16) | 1.06 (0.96, 1.15) |
| **Dampness, all** | **461 (23.5)** | **284.75** | **47.65 (24.79, 70.50)** | **44.41 (21.28, 67.55)** | **1.20 (1.10, 1.31)** | **1.19 (1.09, 1.29)** |
| During last 12 mo | 176 (9.0) | 288.07 | 50.96 (15.99, 85.93) | 51.39 (15.54, 87.23) | 1.21 (1.07, 1.38) | 1.22 (1.07, 1.38) |
| At pregnancy | 38 (1.9) | 307.89 | 70.79 (-5.55, 147.13) | 69.28 (-8.16, 146.72) | 1.30 (1.01, 1.67) | 1.29 (1.00, 1.66) |
| < 1 years | 57 (2.9) | 312.28 | 75.18 (11.97, 138.38) | 72.50 (8.88, 136.12) | 1.32 (1.07, 1.62) | 1.31 (1.06, 1.61) |
| 1-3 years | 98 (5.0) | 315.46 | 78.36 (29.15, 127.57) | 79.25 (29.31, 129.20) | 1.33 (1.14, 1.56) | 1.32 (1.13, 1.56) |
| 4-6 years | 166 (8.5) | 322.89 | 85.79 (46.90, 124.68) | 83.57 (44.60, 122.53) | 1.36 (1.20, 1.54) | 1.36 (1.20, 1.55) |
| 7-9 years | 166 (8.5) | 292.73 | 55.62 (19.57, 91.68) | 50.57 (14.48, 86.67) | 1.23 (1.09, 1.40) | 1.21 (1.07, 1.38) |
| 10-12 years | 77 (3.9) | 287.01 | 49.91 (-1.15, 100.97) | 48.70 (-2.99, 100.39) | 1.21 (1.01, 1.45) | 1.21 (1.01, 1.45) |
| cumulative exposure**^b^** |  |  | **30.91 (16.51, 45.31)** | **28.35 (13.99, 42.72)** | **1.11 (1.06, 1.16)** | **1.10 (1.05, 1.15)** |
| **Visible mold, all** | **213 (10.8)** | **309.43** | 72.33 (38.71, 105.95) | 64.88 (30.59, 99.17) | 1.31 (1.17, 1.46) | 1.27 (1.14, 1.43) |
| During last 12 mo | 72 (3.7) | 336.11 | 99.01 (39.79, 158.22) | 95.25 (34.90, 155.60) | 1.42 (1.19, 1.70) | 1.38 (1.15, 1.66) |
| At pregnancy | 16 (0.8) | 318.75 | 81.65 (-38.09, 201.39) | 77.11 (-41.51, 195.72) | 1.34 (0.92, 1.96) | 1.33 (0.91, 1.94) |
| < 1 years | 28 (1.4) | 314.29 | 77.18 (-12.75, 167.11) | 72.08 (-16.79, 160.95) | 1.33 (0.99, 1.77) | 1.31 (0.98, 1.75) |
| 1-3 years | 50 (2.5) | 328.00 | 90.90 (12.34, 160.45) | 87.93 (18.14, 157.72) | 1.38 (1.12, 1.71) | 1.37 (1.11, 1.71) |
| 4-6 years | 80 (4.1) | 311.25 | 74.15 (20.84, 127.46) | 72.61 (18.21, 127.02) | 1.31 (1.10, 1.56) | 1.30 (1.09, 1.55) |
| 7-9 years | 74 (3.8) | 269.86 | 32.76 (-17.42, 82.93) | 34.78 (-16.04, 85.60) | 1.14 (0.94, 1.37) | 1.15 (0.95, 1.38) |
| 10-12 years | 39 (2.0) | 307.89 | 70.79 (-4.65, 146.23) | 54.87 (-19.44, 129.19) | 1.30 (1.01, 1.66) | 1.24 (0.96, 1.60) |
| cumulative exposure**^b^** |  |  | **27.08 (6.95, 47.21)** | **23.58 (3.47, 43.70)** | **1.09 (1.03, 1.16)** | **1.09 (1.02, 1.16)** |
| **Mold odor, all** | **174 (8.9)** | **324.28** | 87.17 (48.79, 125.56) | 80.62 (41.83, 119.41) | 1.36 (1.21, 1.55) | 1.35 (1.19, 1.53) |
| During last 12 mo | 62 (3.2) | 326.22 | 89.68 (26.28, 151.97) | 83.59 (18.74, 148.45) | 1.38 (1.13, 1.67) | 1.34 (1.09, 1.63) |
| At pregnancy | 10 (0.5) | 450.00 | 212.90 (17.75, 408.04 | 209.60 (13.64, 405.55) | 1.90 (1.23, 2.93) | 1.80 (1.17, 2.79) |
| < 1 years | 22 (1.1) | 377.27 | 140.17 (24.75, 255.59) | 133.95 (19.34, 248.56) | 1.59 (1.17, 2.16) | 1.56 (1.14, 2.12) |
| 1-3 years | 36 (1.8) | 341.67 | 104.56 (20.29, 188.84) | 100.35 (15.79, 184.92) | 1.44 (1.12, 1.85) | 1.42 (1.11, 1.83) |
| 4-6 years | 67 (3.4) | 319.40 | 82.30 (22.66, 141.94) | 77.03 (16.53, 137.54) | 1.35 (1.12, 1.63) | 1.34 (1.10, 1.62) |
| 7-9 years | 56 (2.9) | 281.82 | 44.71 (-14.56, 103.99) | 48.97 (-11.09, 109.04) | 1.19 (0.96, 1.47) | 1.20 (0.97, 1.49) |
| 10-12 years | 32 (1.6) | 287.10 | 49.99 (-29.27, 129.26) | 35.33 (-43.00, 113.65) | 1.21 (0.92, 1.60) | 1.14 (0.85, 1.52) |
| cumulative exposure**^b^** |  |  | **37.45 (12.91, 61.99)** | **34.15 (9.32, 58.97)** | **1.13 (1.05, 1.21)** | **1.12 (1.04, 1.20)** |
| **Any mold exposure, all** | **278 (14.1)** | **310.11** | 73.00 (43.04, 102.97) | 66.78 (36.32, 97.24) | 1.31 (1.18, 1.45) | 1.29 (1.16, 1.43) |
| During last 12 mo | 97 (4.9) | 320.83 | 83.73 (33.93, 133.53) | 80.50 (29.46, 131.54) | 1.35 (1.16, 1.58) | 1.33 (1.13, 1.56) |
| At pregnancy | 22 (1.1) | 327.27 | 90.17 (-13.95, 194.28) | 77.98 (-24.98, 180.94) | 1.38 (1.00, 1.90) | 1.32 (0.96, 1.82) |
| < 1 years | 38 (1.9) | 336.84 | 99.74 (18.50, 180.97) | 86.84 (6.60, 167.08) | 1.42 (1.11, 1.81) | 1.37 (1.07, 1.75) |
| 1-3 years | 63 (3.2) | 319.05 | 81.94 (21.10, 142.79) | 75.97 (15.08, 136.86) | 1.35 (1.11, 1.63) | 1.33 (1.09, 1.61) |
| 4-6 years | 109 (5.5) | 317.43 | 80.33 (33.40, 127.25) | 78.72 (30.52, 126.93) | 1.34 (1.15, 1.56) | 1.33 (1.14, 1.56) |
| 7-9 years | 97 (4.9) | 271.86 | 34.77 (-9.42, 78.96) | 37.06 (-7.66, 81.77) | 1.15 (0.97, 1.35) | 1.16 (0.98, 1.37) |
| 10-12 years | 51 (2.6) | 300.00 | 62.90 (-1.70, 127.49) | 48.84 (-14.72, 112.40) | 1.27 (1.02, 1.57) | 1.21 (0.97, 1.51) |

Abbreviations: CI, confidence interval, IRD, incidence rate difference; IRR, incidence rate ratio, LRTI, lower respiratory infection; NA, not applicable; PY, person-years

^a^Adjusted for sex, family socioeconomic status at baseline, duration of breastfeeding, maternal smoking during pregnancy, and environmental tobacco smoke exposure during pregnancy and from 0 to 3 years of age.

^b^Cumulative exposure per one time period of the six time periods in pregnancy and childhood (during pregnancy, < 1 years, 1-3 years, 4-6 years, 7-9 years, and 10-12 years)

**Table S5. Incidence rates (IR) per 100 person-years (PY), incidence rate differences (IRDs) and incidence rate ratios (IRRs) with 95% confidence intervals of lower respiratory tract infections in the previous 12 months according to the reported exposure to home dampness and molds at 6-year follow-up (before 13 years of age), The Espoo Cohort Study, 1991-2011.**

| **Type of exposure** | **Number (%)** | **IR/100 PY** | **unadjusted IRD (95% CI)** | **adjusted IRD (95% CI) ^a,b^** | **unadjusted IRR (95% CI)** | **adjusted IRR (95% CI)^a^** |
| --- | --- | --- | --- | --- | --- | --- |
| Total N | 1984 | 13.81 |  |  |  |  |
| No exposure | **1395** | **12.08** | 1 (reference) | 1 (reference) | 1 (reference) | 1 (reference) |
| **Any exposure** | **579 (29.3)** | **18.07** | **6.00 (0.82, 11.17)** | **6.73 (0.60, 12.86)** | **1.50 (1.09, 2.06)** | **1.42 (1.02, 1.98)** |
| During last 12 mo | 224 (11.4) | 14.58 | 2.51 (-4.30, 9.32) | 7.71 (-2.98, 18.40) | 1.20 (0.75, 1.95) | 1.16 (0.71, 1.91) |
| At pregnancy | 48 (2.4) | 34.88 | 22.81 (-3.09, 48.70) | 66.79 (-396.77, 530.36) | 2.89 (1.35, 6.20) | 2.95 (1.31, 6.64) |
| < 1 years | 67 (3.4) | 29.51 | 17.43 (-1.90, 36.76) | 56.16 (-132.63, 244.96) | 2.44 (1.24, 4.81) | 2.75 (1.34, 5.68) |
| 1-3 years | 126 (6.4) | 27.78 | 15.70 (1.74, 29.66) | 38.07 (-3.98, 80.12) | 2.30 (1.35, 3.91) | 2.29 (1.31, 3.98) |
| 4-6 years | 217 (11.0) | 22.75 | 10.68 (1.70, 19.65) | 19.49 (5.65, 33.32) | 1.88 (1.23, 2.88) | 1.93 (1.24, 2.99) |
| 7-9 years | 220 (11.1) | 19.55 | 7.48 (-0.96, 15.91) | 14.58 (2.66, 26.49) | 1.62 (1.03, 2.55) | 1.52 (0.94, 2.46) |
| 10-12 years | 95 (4.8) | 12.66 | 0.58 (-8.93, 10.09) | 12.35 (-10.66, 35.37) | 1.05 (0.49, 2.23) | 0.94 (0.41, 2.16) |
| cumulative exposure^c^ |  |  | 4.62 (1.22, 8.02) | 9.11 (3.57, 14.64) | 1.30 (1.12, 1.51) | 1.31 (1.13, 1.53) |
| **Water damage, all** | **186 (9.5)** | **21.51** | **9.44 (-0.03, 18.92)** | **19.56 (0.49, 38.63)** | **1.78 (1.12, 2.84)** | **1.52 (0.93, 2.49)** |
| During last 12 mo | 52 (2.7) | 17.39 | 5.32 (-9.89, 20.52) | NA | 1.44 (0.59, 1.49) | 1.26 (0.51, 3.12) |
| At pregnancy | 8 (0.4) | 33.33 | 21.26 (44.51, 87.02) | NA | 2.76 (0.38, 20.01) | 2.88 (0.39, 21.36) |
| < 1 years | 13 (0.7) | 9.10 | -2.99 (-23.36, 17.39) | NA | 0.75 (0.08, 7.03) | 0.88 (0.09, 8.61) |
| 1-3 years | 32 (1.6) | 15.38 | 3.31 (-15.28, 21.89) | NA | 1.27 (0.38, 4.29) | 1.13 (0.33, 3.92) |
| 4-6 years | 65 (3.3) | 22.64 | 10.57 (-6.09, 27.22) | 49.68 (-51.48, 150.83) | 1.87 (0.88, 3.98) | 2.01 (0.93, 4.35) |
| 7-9 years | 51 (2.6) | 23.08 | 11.00 (-9.08, 31.08) | NA | 1.91 (0.79, 4.63) | 1.23 (0.45, 3.36) |
| 10-12 years | 23 (1.2) | 15.00 | 2.92 (-17.93, 23.78) | NA | 1.24 (0.31, 5.01) | 0.44 (0.05, 3.71) |
| cumulative exposure^c^ |  |  | 5.85 (-2.94, 14.65) | 20.17 (-7.41, 47.74) | 1.36 (0.90, 2.05) | 1.21 (0.79, 1.86) |
| **Dampness, all** | **461 (23.5)** | **18.43** | **6.36 (0.61, 12.11)** | **8.29 (0.88, 15.70)** | **1.53 (1.08, 2.15)** | **1.48 (1.03, 2.11)** |
| During last 12 mo | 176 (9.0) | 15.23 | 3.16 (-4.69, 11.00) | 12.29 (-3.53, 28.11) | 1.26 (0.74, 2.14) | 1.20 (0.69, 2.08) |
| At pregnancy | 38 (1.9) | 30.30 | 18.23 (-8.47, 44.93) | 63.80 (-278.13, 405.73) | 2.51 (1.02, 6.16) | 2.82 (1.07, 7.40) |
| < 1 years | 57 (2.9) | 33.33 | 21.26 (-1.83, 44.35) | 66.03 (-478.45, 610.52) | 2.76 (1.35, 5.64) | 3.13 (1.46, 6.74) |
| 1-3 years | 98 (5.0) | 32.93 | 20.85 (2.76, 38.94) | 52.01 (-14.26, 118.28) | 2.73 (1.53, 4.86) | 2.79 (1.52, 5.13) |
| 4-6 years | 166 (8.5) | 23.29 | 11.21 (0.77, 21.65) | 23.05 (5.48, 40.63) | 1.93 (1.20, 3.11) | 2.02 (1.23, 3.32) |
| 7-9 years | 166 (8.5) | 16.18 | 4.10 (-4.44, 12.64) | 14.12 (-0.12, 28.35) | 1.34 (0.78, 2.30) | 1.36 (0.78, 2.40) |
| 10-12 years | 77 (3.9) | 12.31 | 0.23 (-10.06, 10.52) | 13.91 (-17.43, 45.26) | 1.02 (0.44, 2.35) | 0.86 (0.33, 2.20) |
| cumulative exposure^c^ |  |  | 4.30 (4.30, 4.30) | 10.23 (3.07, 17.39) | 1.28 (1.08, 1.51) | 1.31 (1.11, 1.56) |
| **Visible mold, all** | **213 (10.8)** | **21.62** | **9.55 (0.50, 18.59)** | **18.66 (2.36, 34.96)** | **1.79 (1.14, 2.80)** | **1.70 (1.06, 2.74)** |
| During last 12 mo | 72 (3.7) | 12.07 | -0.01 (-10.76, 10.74) | 21.60 (-37.21, 80.40) | 1.00 (0.41, 2.44) | 1.06 (0.43, 2.66) |
| At pregnancy | 16 (0.8) | 7.69 | -4.38 (-21.37, 12.60) | NA | 0.64 (0.07, 5.73) | 0.71 (0.08, 6.54) |
| < 1 years | 28 (1.4) | 16.67 | 4.59 (-15.59, 24.77) | NA | 1.38 (0.41, 4.66) | 1.62 (0.46, 5.68) |
| 1-3 years | 50 (2.5) | 38.64 | 26.56 (-0.49, 53.61) | 78.28 (-78.76, 228.11) | 3.20 (1.55, 6.59) | 3.01 (1.40, 6.42) |
| 4-6 years | 80 (4.1) | 24.29 | 12.21 (-3.33, 27.75) | 34.57 (-11.79, 80.94) | 2.01 (1.04, 3.89) | 2.01 (0.98, 4.09) |
| 7-9 years | 74 (3.8) | 17.86 | 5.78 (-8.20, 19.76) | 42.04 (-130.63, 214.71) | 1.48 (0.67, 3.28) | 1.47 (0.63, 3.43) |
| 10-12 years | 39 (2.0) | 16.67 | 4.59 (-13.61, 22.80) | NA | 1.38 (0.56, 4.15) | 1.03 (0.27, 3.96) |
| cumulative exposure^c^ |  |  | 6.74 (-0.56, 14.04) | 13.15 (1.12, 25.18) | 1.32 (1.02, 1.72) | 1.31 (1.00, 1.72) |
| **Mold odor, all** | **174 (8.9)** | **21.71** | **9.36 (-0.28, 19.55)** | **25.84 (-0.71, 52.40)** | **1.80 (1.11, 2.92)** | **1.75 (1.06, 2.90)** |
| During last 12 mo | 62 (3.2) | 13.46 | 1.39 (10.72, 13.49) | 32.54 (-125.44, 190.52) | 1.11 (0.45, 2.75) | 1.16 (0.45, 2.96) |
| At pregnancy | 10 (0.5) | 33.33 | NA | NA | 2.76 (0.52, 14.54) | 1.33 (0.20, 8.81) |
| < 1 years | 22 (1.1) | 25.00 | 12.92 (16.75, 42.60) | NA | 2.07 (0.62, 6.87) | 1.91 (0.52, 6.93) |
| 1-3 years | 36 (1.8) | 40.63 | 28.55 (-5.15, 62.25) | NA | 3.36 (1.44, 7.87) | 3.19 (1.30, 7.78) |
| 4-6 years | 67 (3.4) | 20.97 | 8.89 (-5.73, 23.52) | 45.26 (-37.03, 127.55) | 1.74 (0.85, 3.55) | 1.91 (0.92, 4.01) |
| 7-9 years | 56 (2.9) | 26.19 | 14.11 (-6.79, 35.02) | NA | 2.17 (0.96, 4.90) | 2.42 (1.04, 5.63) |
| 10-12 years | 32 (1.6) | 14.29 | 2.21 (-12.01, 16.42) | NA | 1.18 (0.36, 3.93) | 0.67 (0.14, 3.22) |
| cumulative exposure^c^ |  |  | 7.06 (-0.80, 14.92) | 14.90 (0.07, 29.73) | 1.35 (1.04, 1.77) | 1.36 (1.03, 1.78) |
| **Any mold exposure, all** | **278 (14.1)** | **19.59** | **7.52 (0.03, 15.00)** | **13.45 (2.05, 24.85)** | **1.62 (1.08, 2.45)** | **1.56 (1.02, 2.40)** |
| During last 12 mo | 97 (4.9) | 11.25 | -0.83 (-9.66, 8.01) | 14.53 (-11.85, 40.90) | 0.93 (0.43, 2.04) | 1.00 (0.45, 2.25) |
| At pregnancy | 22 (1.1) | 21.05 | 8.98 (-18.12, 36.07) | NA | 1.74 (0.48, 6.38) | 1.26 (0.31, 5.14) |
| < 1 years | 38 (1.9) | 20.59 | 8.51 (-6.99, 24.01) | 71.52 (71.52, 71.52) | 1.70 (0.64, 4.52) | 1.64 (0.59, 4.58) |
| 1-3 years | 63 (3.2) | 31.58 | 19.50 (-1.08, 40.09) | 58.46 (-43.36, 162.28) | 2.62 (1.33, 5.14) | 2.43 (1.20, 4.91) |
| 4-6 years | 109 (5.5) | 23.47 | 11.39 (-1.38, 24.17) | 39.77 (-4.83 66.37) | 1.94 (1.10, 3.43) | 1.97 (1.08, 3.61) |
| 7-9 years | 97 (4.9) | 18.67 | 6.59 (-5.88, 19.06) | 32.83 (-27.83, 93.50) | 1.55 (0.78, 3.06) | 1.57 (0.76, 3.22) |
| 10-12 years | 51 (2.6) | 12.20 | 0.12 (-12.70, 12.94) | NA | 1.01 (0.35, 2.89) | 0.70 (0.19, 2.53) |

Abbreviations: CI, confidence interval, IRD, incidence rate difference; IRR, incidence rate ratio, LRTI, lower respiratory infection; NA, not applicable; PY, person-years

^a^Adjusted for sex, family socioeconomic status at baseline, duration of breastfeeding, maternal smoking during pregnancy, and environmental tobacco smoke exposure during pregnancy and from 0 to 3 years of age.

^b^ Unstable estimates are likely to due to sparce data with small numbers of observations.

^c^ Cumulative exposure per one time period of the six time periods in pregnancy and childhood (during pregnancy, < 1 years, 1-3 years, 4-6 years, 7-9 years, and 10-12 years)

# Home dampness and molds and respiratory tract infections at 20-year follow-up

**Table S6. Incidence rates (IR) per 100 person-years (PY), incidence rate differences (IRDs) and incidence rate ratios (IRRs) with 95% confidence intervals of upper respiratory tract infections in the previous 12 months according to the reported exposure to home dampness and molds at 20-year follow-up (before 27 years of age), The Espoo Cohort Study, 1991-2011.**

| **Type of exposure** | **Number (%)** | **IR/100 PY** | **unadjusted IRD (95% CI)** | **adjusted IRD (95% CI)** ^a^ | **unadjusted IRR (95% CI)** | **adjusted IRR (95% CI) ^a^** |
| --- | --- | --- | --- | --- | --- | --- |
| Total N | 1613 | 265.10 |  |  |  |  |
| **No exposure** | 1285 (79.7) | 255.55 | 1 (reference) | 1 (reference) | 1 (reference) | 1 (reference) |
| **Any exposure** | **328 (20.3)** | 301.26 | **45.71** (**18.23, 73.20**) | **39.99 (12.72, 67.27)** | **1.18 (1.07, 1.30**) | **1.15 (1.05, 1.26)** |
| Last 12 months | 203 | 314.72 | 59.17 (24.90, 93.44) | 48.40 (14.78, 82.01) | 1.23 (1.10, 1.38) | 1.18 (1.05, 1.32) |
| Over 12 months ago | 125 | 300.68 | 45.13 (6.68, 83.58) | 43.53 (4.77, 82.29) | 1.18 (1.03, 1.34) | 1.16 (1.02, 1.33) |
| **Water damage** | **116 (7.25)** | 314.41 | **58.87** (**14.15, 103.59**) | **57.14 (13.19, 101.09)** | **1.23 (1.06, 1.42**) | **1.24 (1.07, 1.43)** |
| Last 12 months | 40 | 334.21 | 78.66 (1.80, 155.53) | 78.19 (3.37, 153.02) | 1.31 (1.04, 1.65) | 1.32 (1.05, 1.65) |
| Over 12 months ago | 76 | 304.11 | 48.56 (-4.75, 101.87) | 47.27 (-5.25, 99.79) | 1.19 (1.00, 1.42) | 1.20 (1.00, 1.43) |
| **Dampness** | **222 (14.7)** | 310.91 | **55.36** (**22.74, 87.99**) | **47.83 (15.35, 80.31)** | **1.22 (1.09, 1.36**) | **1.17 (1.05, 1.31)** |
| Last 12 months | 137 | 321.80 | 66.26 (25.12, 107.40) | 51.37 (11.17, 91.58) | 1.26 (1.11, 1.43) | 1.18 (1.03, 1.35) |
| Over 12 months ago | 88 | 294.25 | 38.71 (-9.49, 86.91) | 42.55 (-6.65, 91.75) | 1.15 (0.98, 1.36) | 1.15 (0.98, 1.36) |
| **Visible mold** | **59 (3.68)** | 291.52 | **35.98** (**-20.58, 92.54**) | **32.47 (-24.99, 89.92)** | **1.**14 (**0.94, 1.39**) | **1.11 (0.91, 1.35)** |
| Last 12 months | 37 | 340.54 | 84.99 (6.26, 163.72) | 77.33 (-0.30, 154.96) | 1.33 (1.06, 1.68) | 1.27 (1.01, 1.60) |
| Over 12 months ago | 22 | 209.09 | -46.46 (-119.86, 26.95) | -50.38 (-127.52, 26.76) | 0.81 (0.58, 1.16) | 0.80 (0.56, 1.15) |
| **Mold odor** | **83 (5.17)** | 317.28 | **61.74** (**9.74, 113.74**) | **49.37 (-1.56, 100.21)** | **1.**24 (1**.05, 1.47**) | **1.18 (1.00, 1.40)** |
| Last 12 months | 62 | 325.00 | 69.45 (8.12, 130.79) | 53.11 (-6.22, 112.44) | 1.27 (1.05, 1.54) | 1.19 (0.98, 1.45) |
| Over 12 months ago | 21 | 295.23 | 39.69 (-54.24, 133.62) | 39.97 (-54.42, 134.36) | 1.16 (0.84, 1.59) | 1.15 (0.83, 1.58) |
| **Any mold exposure** | **119 (7.5)** | 300.84 | **45.30** (**3.31, 87.30**) | **35.42 (-6.29, 77.14)** | **1.18 (1.02, 1.36**) | **1.13 (0.98, 1.30)** |
| Last 12 months | 87 | 324.71 | 69.16 (17.49, 120.83) | 53.42 (3.20, 103.64) | 1.27 (1.08, 1.49) | 1.20 (1.02, 1.41) |
| Over 12 months ago | 33 | 245.71 | -9.83 (-74.64, 54.98) | -8.03 (-75.41, 59.35) | 0.96 (0.74, 1.25) | 0.95 (0.73, 1.25) |

Abbreviations: CI, confidence interval, IRD, incidence rate difference; IRR, incidence rate ratio, URTI, upper respiratory infection; PY, person-years

^a^Adjusted for sex, family socioeconomic status at baseline, duration of breastfeeding, maternal smoking during pregnancy, and environmental tobacco smoke exposure during pregnancy and from 0 to 3 years of age.

**Table S7. Incidence rates (IR) per 100 person-years (PY), incidence rate differences (IRDs) and incidence rate ratios (IRRs) with 95% confidence intervals of lower respiratory tract infections in the previous 12 months according to the reported exposure to home dampness and molds at 20-year follow-up (before 27 years of age), The Espoo Cohort Study, 1991-2011.**

| **Type of exposure** | **Number (%)** | **IR/100 PY** | **unadjusted IRD (95% CI)** | **adjusted IRD (95% CI)**^a^ | **unadjusted IRR (95% CI)** | **adjusted IRR (95% CI) ^a^** |
| --- | --- | --- | --- | --- | --- | --- |
| Total N | 1613 | 12.88 |  |  |  |  |
| **No exposure** | 1285 (79.7) | 10.44 | 1 (reference) | 1 (reference) | 1 (reference) | 1 (reference) |
| **Any exposure** | **328 (20.3)** | 22.64 | **12.20** (**3.48, 20.92**) | **12.14 (3.60, 20.67)** | **2.17** (**1.40, 3.35**) | **2.15 (1.37, 3.37)** |
| Last 12 months | 203 | 21.32 | 10.88 (0.16, 21.60) | 9.82 (-0.05, 19.69) | 2.04 (1.19, 3.51) | 1.88 (1.07, 3.28) |
| Over 12 months ago | 125 | 31.97 | 21.53 (5.07, 37.99) | 23.59 (6.80, 40.39) | 3.06 (1.75, 5.35) | 3.43 (1.93, 6.10) |
| **Water damage** | **116 (7.25)** | 36.04 | **25.60** (**3.80, 47.39**) | **28.15 (6.19, 50.11)** | **3.45** (**1.81, 6.58**) | **3.94 (2.04, 7.60)** |
| Last 12 months | 40 | 13.16 | 2.72 (-13.90, 19.34) | 6.26 (-11.32, 23.83) | 1.26 (0.35, 4.49) | 1.42 (0.41, 4.90) |
| Over 12 months ago | 76 | 47.95 | 37.50 (3.74, 71.27) | 40.12 (6.28, 73.96) | 4.59 (2.19, 9.62) | 5.46 (2.58, 11.55) |
| **Dampness** | **222 (14.7)** | 21.82 | **11.38** (**1.41, 21.34**) | **11.82 (2.04, 21.59)** | **2.09** (**1.27, 3.44**) | **2.09 (1.25, 3.50)** |
| Last 12 months | 137 | 23.31 | 12.87 (-1.06, 26.80) | 12.78 (-0.48, 26.04) | 2.23 (1.19, 4.20) | 2.20 (1.15, 4.21) |
| Over 12 months ago | 88 | 19.54 | 9.10 (-5.08, 23.28) | 9.88 (-4.36, 24.12) | 1.87 (0.88, 3.96) | 1.95 (0.91, 4.16) |
| **Visible mold** | **59 (3.68)** | 28.81 | **18.37** (**-6.94, 43.69**) | **20.78 (-5.04, 46.60)** | **2.76** (**1.12, 6.83**) | **3.01 (1.21, 7.52)** |
| Last 12 months | 37 | 40.54 | 30.10 (-12.69, 72.89) | 34.19 (-8.75, 77.12) | 3.88 (1.32, 11.43) | 4.57 (1.59, 13.14) |
| Over 12 months ago | 22 | 9.09 | -1.35 (-17.90, 15.20) | -6.22 (-103.66, 91.22) | 0.87 (0.14, 5.35) | 0.40 (0.04, 3.98) |
| **Mold odor** | **83 (5.17)** | 40.74 | **30.30** (**2.80, 57.80**) | **28.48 (2.91, 54.06)** | **3.90** (**1.92, 7.94**) | **3.81 (1.83, 7.90)** |
| Last 12 months | 62 | 45.00 | 34.56 (-0.73, 69.85) | 30.76 (-1.03, 62.55) | 4.31 (1.91, 9.74) | 3.97 (1.72, 9.18) |
| Over 12 months ago | 21 | 28.57 | 18.01 (-22.07, 58.08) | 22.19 (-19.69, 64.08) | NA | 3.39 (0.83, 13.78) |
| **Any mold exposure** | **119 (7.5)** | 33.90 | **23.46** (**4.27, 42.64**) | **21.82 (3.83, 39.81)** | **3.25** (**1.77, 5.95**) | **3.12 (1.66, 5.86)** |
| Last 12 months | 87 | 37.65 | 27.21 (2.05, 52.36) | 24.42 (1.69, 47.14) | 3.61 (1.78, 7.29) | 3.40 (1.65, 6.97) |
| Over 12 months ago | 33 | 22.86 | 12.42 (-13.43, 38.27) | 12.97 (-12.64, 38.59) | 2.19 (0.69, 6.91) | 2.23 (0.68, 7.34) |

Abbreviations: CI, confidence interval, IRD, incidence rate difference; IRR, incidence rate ratio, NA, not applicable; LRTI, lower respiratory infection; PY, person-years

^a^Adjusted for sex, family socioeconomic status at baseline, duration of breastfeeding, maternal smoking during pregnancy, and environmental tobacco smoke exposure during pregnancy and from 0 to 3 years of age.

# Home dampness and molds and respiratory tract infections based on the hospitalization register

**Table S8. Incidence rates (IR) per 100 person-years (PY), IRD and IRR with 95% confidence intervals of upper respiratory tract infections (URTI) requiring hospitalization or health care visit according to the reported exposure to home dampness and molds, The Espoo Cohort Study, 1991-2011.**

|  | **IR/100 person-years** | **unadjusted IRD (95% CI)** | **adjusted IRD (95% CI) ^a^** | **unadjusted IRR (95% CI)** | **adjusted IRR (95% CI) ^a^** |
| --- | --- | --- | --- | --- | --- |
| **URTI at 0-6 years (6 person-years)** |  |  |  |  |  |
| No exposure (ref) | 8.76 |  |  |  |  |
| Any exposure, baseline | 11.38 | 3.23 (-1.42, 7.87) | 2.38 (-1.85, 6.62) | 1.40 (0.91, 2.15) | 1.40 (0.90, 2.19) |
| **URTI at 7-13 years (7 person-years)** |  |  |  |  |  |
| No exposure (ref) | 2.06 |  |  |  |  |
| Any exposure, baseline | 1.62 | -0.55 (-2.28, 1.19) | NA | 0.75 (0.27, 2.04) | 0.72 (0.24, 2.10) |
| Any exposure, 6-year follow-up | 3.28 | 1.92 (-0.56, 4.40) | NA | 2.41 (0.96, 6.05) | 1.79 (0.65, 4.94) |
| **URTI at 14-27 years (14 person-years)** |  |  |  |  |  |
| No exposure (ref) | 9.23 |  |  |  |  |
| Any exposure, baseline | 16.67 | 9.19 (1.40, 16.97) | 14.24 (4.83, 23.65) | 2.23 (1.33, 3.74) | 2.26 (1.27, 4.00) |
| Any exposure, 6-year follow-up | 8.29 | -1.67 (-6.77, 3.43) | -0.28 (-3.59, 3.03) | 0.83 (0.47, 1.49) | 0.87 (0.48, 1.58) |
| Any exposure, 20-year follow-up | 9.76 | 0.88 (-6.10, 7.87) | 14.61 (-4.58, 33.81) | 1.10 (0.53, 2.28) | 1.42 (0.66, 3.07) |
| **URTI at 0-27 years (27 person-years)** |  |  |  |  |  |
| No exposure (ref) | 20.05 |  |  |  |  |
| Any exposure, baseline | 29.67 | 11.87 (3.04, 20.69) | 13.08 (2.90, 23.25) | 1.67 (1.21, 2.30) | 1.71 (1.22, 2.41) |

Abbreviations: CI, confidence interval; IR, incidence rate; IRD, incidence rate difference; IRR, incidence rate ratio, NA, not applicable; URTI, upper respiratory infection

^a^Adjusted for sex, family socioeconomic status at baseline, duration of breastfeeding, maternal smoking during pregnancy, and environmental tobacco smoke exposure during pregnancy and from 0 to 3 years of age.

**Table S9. Incidence rates (IR) per 100 person-years (PY), IRD and IRR with 95% confidence intervals of lower respiratory tract infections (LRTI) hospitalization or health care visit according to the reported exposure to home dampness and molds, The Espoo Cohort Study, 1991-2011.**

|  | **IR/100 person-years** | **unadjusted IRD (95% CI)** | **adjusted IRD (95% CI) ^a^** | **unadjusted IRR (95% CI)** | **adjusted IRR (95% CI) ^a^** |
| --- | --- | --- | --- | --- | --- |
| **LRTI at 0-6 years (6 person-years)** |  |  |  |  |  |
| No exposure (ref) | 4.48 | 1 | 1 | 1 | 1 |
| Any exposure, baseline | 4.67 | 0.23 (-2.67, 3.14) | 1.79 (-0.59, 4.17) | 1.05 (0.56, 1.97) | 1.12 (0.59, 2.12) |
| **LRTIs at 7-13 years (7 person-years)** |  |  |  |  |  |
| No exposure (ref) | 1.05 | 1 | 1 | 1 | 1 |
| Any exposure, baseline | 0.41 | -0.80 (-1.70, 0.10) | NA | 0.34 (0.06, 1.75) | NA |
| Any exposure, 6-year follow-up | 1.38 | 0.23 (-0.89, 1.36) | NA | 1.20 (0.37, 3.96) | 1.07 (0.29, 3.95) |
| **LRTIs at 14-27 years (14 person-years)** |  |  |  |  |  |
| No exposure (ref) | 3.19 | 1 | 1 | 1 | 1 |
| Any exposure, baseline | 3.56 | 0.32 (-3.35, 3.98) | NA | 1.10 (0.37, 3.24) | NA |
| Any exposure, 6-year | 3.97 | 1.61 (-2.17, 5.39) | NA | 1.68 (0.58, 4.89) | 1.54 (0.51, 4.58) |
| Any exposure, 20-year | 4.27 | 1.78 (-3.91, 7.47) | NA | 1.71 (0.40, 7.26) | 2.27 (0.52, 10.03) |
| **LRTIs at 0-27 years (27 person-years)** |  |  |  |  |  |
| No exposure (ref) | 8.72 | 1 | 1 | 1 | 1 |
| Any exposure, baseline | 8.54 | -0.25 (-4.72, 4.23) | 1.34 (-2.93, 5.60) | 0.97 (0.58, 1.64) | 0.88 (0.27, 2.89) |

Abbreviations: CI, confidence interval; IR, incidence rate; IRD, incidence rate difference; IRR, incidence rate ratio, NA, not applicable; LRTI, lower respiratory infection

^a^Adjusted for sex, family socioeconomic status at baseline, duration of breastfeeding, maternal smoking during pregnancy, and environmental tobacco smoke exposure during pregnancy and from 0 to 3 years of age
